# The Changes in Endogenous Metabolites in Hyperlipidemic Rats Treated with Herbal Mixture Containing Lemon, Apple Cider, Garlic, Ginger, and Honey

**DOI:** 10.3390/nu13103573

**Published:** 2021-10-12

**Authors:** Azliana Abu Bakar Sajak, Azrina Azlan, Faridah Abas, Hazilawati Hamzah

**Affiliations:** 1 Department of Nutrition, Faculty of Medicine and Health Sciences, Universiti Putra Malaysia (UPM), Serdang 43400, Selangor, Malaysia; azleeyana@yahoo.com; 2 Research Centre for Excellence for Nutrition and Non-Communicable Disease, Faculty of Medicine and Health Sciences, Universiti Putra Malaysia (UPM), Serdang 43400, Selangor, Malaysia; 3 Laboratory of Natural Products, Institute of Bioscience, Universiti Putra Malaysia (UPM), Serdang 43400, Selangor, Malaysia; faridah_abas@upm.edu.my; 4 Department of Veterinary Medicine, Faculty of Veterinary Medicine, Universiti Putra Malaysia (UPM), Serdang 43400, Selangor, Malaysia; hazilawati@upm.edu.my

**Keywords:** herbal, anti-hyperlipidemic, urine metabolomics, metabolites, hydroxymethylglutaryl-CoA reductase

## Abstract

An herbal mixture composed of lemon, apple cider, garlic, ginger and honey as a polyphenol-rich mixture (PRM) has been reported to contain hypolipidemic activity on human subjects and hyperlipidemic rats. However, the therapeutic effects of PRM on metabolites are not clearly understood. Therefore, this study aimed to provide new information on the causal impact of PRM on the endogenous metabolites, pathways and serum biochemistry. Serum samples of hyperlipidemic rats treated with PRM were subjected to biochemistry (lipid and liver profile) and hydroxymethylglutaryl-CoA enzyme reductase (HMG-CoA reductase) analyses. In contrast, the urine samples were subjected to urine metabolomics using ^1^H NMR. The serum biochemistry revealed that PRM at 500 mg/kg (PRM-H) managed to lower the total cholesterol level and low-density lipoprotein (LDL-C) (*p* < 0.05) and reduce the HMG-CoA reductase activity. The pathway analysis from urine metabolomics reveals that PRM-H altered 17 pathways, with the TCA cycle having the highest impact (0.26). Results also showed the relationship between the serum biochemistry of LDL-C and HMG-CoA reductase and urine metabolites (trimethylamine-*N*-oxide, dimethylglycine, allantoin and succinate). The study’s findings demonstrated the potential of PRM at 500 mg/kg as an anti-hyperlipidemic by altering the TCA cycle, inhibiting HMG-CoA reductase and lowering the LDL-C in high cholesterol rats.

## 1. Introduction

Hyperlipidemia can be defined as elevated lipid level compared to the normal range, whereby the individual might have an abnormal level (high level) of triglyceride (>200 mg/dL), total cholesterol (≥240 mg/dL) and low-density lipoprotein (LDL, >130 mg/dL) and lower high-density lipoprotein (HDL, <40 mg/dL) [1,2]. It is a well-documented risk factor for cardiovascular disease (CVD), which accounted for 17.9 million deaths in 2016 [2,3]. The primary approach by the physician in dealing with hyperlipidemia focuses on modifying the lifestyle and diet. Nonetheless, in certain chronic cases, the usage of drugs such as statins is preferred in treating hyperlipidemia [2]. However, several side effects of statins, such as muscle pain, digestive problems, nausea, and myopathy, have been reported [4]. Thus, some patients intolerant to statins have opted for herbal medicines as alternatives, since they are considered safe and well-tolerated [5].

For a long time, herbal medicines, such as mixtures containing crude plants, have been used to treat and prevent hyperlipidemia [6]. Plants such as ginseng, ginger, turmeric and lotus jujube have been traditionally used and proven in either preclinical or clinical trials or both [7,8,9,10,11]. According to Ji et al. [12], most herbal medicines intervene and improve the lipid metabolism by inhibiting cholesterol absorption in enterocytes, stimulating reverse transport in multiple organ pathways and regulating cholesterol synthesis and excretion. However, only a few of these herbal remedies are being scientifically evaluated in a thorough manner [13,14]. The usage of a single herb or a mixture of herbs can accumulate from hundreds to thousands of metabolites in a single dose [6]. Due to the wide variety of bioactive compounds, identifying altered pathways and mechanisms of action can be a pretty challenging process.

Recently, an herbal mixture containing common household ingredients such as lemon, ginger, garlic, apple cider vinegar and honey, named a polyphenol-rich extract (PRM), has been hailed as one of the nutritional beverages that reduces cholesterol levels. Some previous studies showed promising effects of the mixture in improving the lipid profile in hyperlipidemia-induced animal models when orally fed or incorporated in the diet [15,16]. It also showed a good result in delaying and lowering postprandial glucose when administered alone or accompanied by exercise in non-diabetic human females after a high carbohydrate meal compared to the control group [17]. Ishak et al. [17] noted the glucose changes were 8% in the mixture group, 13% in the exercise group and 15% in the mixture accompanied with the exercise group. Despite some promising results, the mechanisms that underlay the activities remain unclear.

The advancement of current technologies, especially in analytical instruments, allows the researcher to generate comprehensive metadata in a high throughput manner [18]. However, dealing with metadata containing hundreds to thousands of variables and variations might be overwhelming. With the help of pattern recognition techniques used by multivariate analysis (MVDA), these metadata can be explained in simplified manner for a better understanding.

In recent years, metabolomic or metabonomic studies have emerged for the detection of metabolite changes in organisms. The conventional analytical instruments for the detection are mass spectrophotometer (MS) or nuclear magnetic resonance (NMR). NMR metabolomics has been successfully applied in analyzing complex sample matrices in food chemistry, toxicology and environmental studies [19,20]. It is also being used in the medicinal and pharmacological fields in detecting putative biomarkers, especially biomarkers related to the pathogenesis of the disease and testing for a new lead compound [19]. The information generated from these data can help clinicians and researchers understand the efficacy of the treatment of the disease. In this study, the therapeutic effect of PRM was evaluated using ^1^H-NMR urine metabolomics in hyperlipidemic rats. Thus, this study aimed (1) to identify the metabolites and pathways attributed to the PRM activities and (2) to improve the understanding of the PRM mechanism as an anti-hyperlipidemic agent.

## 2. Materials and Methods

### 2.1. Chemicals and Reagents

The NMR reagents such as deuterated oxide (D_2_O), trimethylsilyl propionic acid-d4 sodium salt (TSP) and sodium deuterium oxide (NaOD) were purchased from Cambridge Isotope Laboratories (Tokyo, Japan). In addition, the non-deuterated KH_2_PO_4_ was purchased from Merck (Darmstadt, Germany). For high-performance liquid chromatography (HPLC) purposes, the chemicals used were high-performance liquid chromatography grade from Fischer Chemicals (Pittsburgh, PA, USA).

### 2.2. Sample Preparation

Polyphenol rich mixture (PRM) preparation was conducted based on our previously published article by Abu Bakar Sajak et al. [21]. In brief, a volume of 1000 mL of juice mixture consisting of lemon (*Citrus limon*), garlic (*Allium sativum*), ginger (*Zingiber officinale*) and apple cider (250 mL each) was simmered at 85 °C for 30 min until it reduced to 750 mL. After being cooled, 750 mL of Tualang honey was added to the mixture. The final mix was kept at 4 °C before use.

### 2.3. Ascorbic Acid (Vitamin C) Determination

The ascorbic content in PRM was determined based on the standard procedure by AOAC International, AOAC 967.21 [22]. In brief, 90 mL of 3% of metaphosphoric acid was added to 10 mL of PRM solution and filtered with Whatman no.1 filter paper to remove any insoluble residue. The filtered mixture (5 mL) was then titrated with 2, 6-dichlorophenol iodophenol (DCPIP) as an indicator for the endpoint. Ascorbic was expressed as milligrams of ascorbic acid per 100 mL of sample. 

### 2.4. Phenolic Extraction Using Solid-Phase Extraction (SPE) and Liquid-Liquid Extraction (LLE)

A solid-phase extraction (SPE) was done on the PRM using the modified method by Jasicka-Misiak et al. [23]. Briefly, a 10 g sample of PRM was dissolved in 50 mL acidified water (pH = 2 with HCl) and homogenized for 60 min using an ultrasonic bath. Next, any solid particle was filtered and removed using Whatman no.1 before proceeding with SPE using strata^®^ SDBL 100 µm styrene-divinylbenzene, 200 mg/6 mL (Phenomenex). For the conditioning step, the cartridge was first washed with methanol. The sample was then loaded to the cartridge and washed with acidified water (0.05 M HCl, 25 mL) and distilled water (30 mL). The compounds of interest were eluted using 20 mL of 50% acidified methanol (0.05 M HCl) and 20 mL of methanol at a 2 mL/min flow rate. The methanol extract was then concentrated under reduced pressure of 40 °C. Next, the dried residue was dissolved in distilled water and underwent liquid–liquid extraction with diethyl ether (15 mL). The diethyl layer was removed, and the remaining aqueous layer was then partitioned with ethyl acetate 3 times (5 mL each). The upper layer of ethyl acetate and the lower aqueous layer were collected. The samples were then concentrated and kept at 4 °C before being analyzed. Chromatographic separation was performed using HPLC with diode array detection (DAD) for PRM in acidified water (without SPE), PRM after SPE, PRM ethyl acetate fraction and PRM aqueous fraction. 

### 2.5. High-Performance Liquid Chromatography (HPLC) Analysis

Chromatographic separation was performed using an Agilent 1100 series (Agilent Technologies, Germany) chromatograph equipped with a binary pump, automatic sampler, degasser and diode-array detector (DAD) and reversed-phase column, Luna C18 (250 × 4.6 mm ID; particle size 5 μm) maintained at 40 °C. The UV was recorded in three channels (214, 280 and 340 nm), and the wavelength of 280 nm was picked for quantification purposes. The two mobile phases consisted of (A) 0.1% formic acid in water and (B) 0.1% methanol in water. The separation of compounds was carried out in gradient elution as follows (A: B ratio in percentage): 80–20% at 0 min, 50–50% at 10 min, 30–70% at 25 min, 10–90% at 30 min, 0–100% at 35 min and 80–20% at 40 min. The entire run was 40 min at a 1 mL/min flow rate, and the injection volume was set to 20 µL. 

The identification of markers present in the PRM extract was based on comparing UV spectra and retention times with those of the standard compounds and spiking the standards inside the PRM. The preparation of fraction and standard (200 µg/mL) was prepared by dissolving it in HPLC grade methanol. The standard curve equations with regression values for markers were: 5-Hydroxymethylfurfural (y = 174.8x, R^2^ = 0.99), 6-shogaol (y = 7.7903x, R^2^ = 0.99), 6-gingerol (y = 10.987x, R^2^ = 0.99).

### 2.6. Liquid Chromatography-Mass Spectroscopy (LC-MS)

LC-MS analysis was performed for PRM after SPE sample to detect the compounds in the PRM. The LC was performed using an Agilent 1290 Infinity LC system coupled with an Agilent 6520 Accurate-Mass Q-TOF mass spectrometer with a dual ESI source. The compounds were chromatographically separated using a column ZORAX Eclipse Plus C18 Rapid Resolution HT (2.1 × 100 mm) and maintained at 40 °C. The linear binary gradient of water contained 0.1% formic acid (mobile phase A) and acetonitrile contained 0.1% formic acid (mobile phase B). The mobile phase composition was changed during the run as follows: 0 min, 5% B; 36 min, 95% B; 41 min, 95% B; 41.10 min, 5% B; 48.00 min, 5% B. The flow rate was set to 0.25 mL/min, and the injection volume was 2 μL. The ion source was operated in positive electrospray ionization (ESI) mode under the following specific conditions: capillary voltage, ±4 kV; heated capillary temperature 325 °C. The total ion chromatograms (TIC) were recorded for m/z 100–m/z 1100. The reference masses were 112.986 and 1033.988. Tentative identification of compounds was made using mass bank [24] and in house database. Only tentative compounds with molecular features that matched more than 80% were chosen.

### 2.7. Efficacy Study on an Animal Model

#### 2.7.1. Acclimatization

Animal study ethics approval was obtained from the Institutional Animal Care and Use Committee (IACUC) Universiti Putra Malaysia (IACUC No. AUP-R012/2017). This efficacy study was conducted in a cleanroom facility at the Comparative Medicine and Technology Unit (COMeT), Universiti Putra Malaysia. A total of forty-two male-specific pathogen-free (SPF) Wistar rats (*n* = 42), five weeks old, weighing 100–150 g, were used in this study. The rats were acclimatized for two weeks upon arrival and placed in an individual ventilated cage (IVC) at the room temperature 25–28 °C, 12 light/dark cycle and ad libitum on standard diet and water.

#### 2.7.2. Hyperlipidemia Induction

After two weeks of acclimatization, hyperlipidemia was induced in five out of six groups of rats. The normal/control group was fed with standard rat chow, while other groups were fed with a high cholesterol diet. The normal diet/standard diet contained 14% fat, 61% carbohydrates and 25% protein while the high-cholesterol diet (HC) consisted of 76% of standard diet, 15% fats, 4% cholesterol, 4% sucrose, 0.8% bile salt and 0.2% propylthiouracil. These diets were given throughout the study period (8 weeks), even after the induction period at week 4.

#### 2.7.3. Treatment with the Polyphenol-Rich Mixture (PRM) and Simvastatin

After hyperlipidemia was successfully induced in the rats, the rats were divided into six groups as follows:

Group C: Normal/Control diet group, given 1 mL of filtered water
Group HC: High cholesterol diet group, given 1 mL of filtered water
Group PRM-H: High cholesterol diet group, given 500 mg/kg PRM
Group PRM-M: High cholesterol diet group, given 250 mg/kg PRM
Group PRM-L: High cholesterol diet group, given 150 mg/kg PRM
Group D: High cholesterol diet group, given simvastatin 10 mg/kg

By the end of week 8, rats were sacrificed by exsanguination under ketamine/xylazine. The blood samples were withdrawn via cardiac puncture. The final number of rats for each group by the end of the efficacy test for C was *n* = 4, HC was *n* = 4, D was *n* = 6, PRM-H was *n* = 7, PRM-M was *n* = 6 and PRM-L was *n* = 6.

#### 2.7.4. Blood and Urine Collection

Blood collection was made on week 4 (after induction of hyperlipidemia) and by the end of week 8 (after treatment). The blood sample was withdrawn from each rat via cardiac puncture and collected into a sterile tube after anaesthetizing with ketamine/xylazine. The serum was separated from the blood sample by centrifugation at high speed for 6000× *g* for 10 min at 4 °C. For urine collection, the collection was made on week four, and at the end of week eight after treatment completion. The rats were placed individually in a plastic metabolic cage equipped with a urine collection bottle added with 0.1% sodium azide. All the biochemical samples were kept at −80 °C until further use.

#### 2.7.5. Serum Biochemistry Analyses and HMG-CoA Reductase

Serum lipid profile (total cholesterol (TC), triglyceride (TG), high-density lipoprotein (HDL-C), low-density lipoprotein (LDL-C)) and liver profile (aspartate transaminase (AST) and alanine aminotransferase (ALT)) were measured using automated biochemical analyzer Hitachi 902 (Roche Diagnostics, Mannheim, Germany). In addition, HMG-CoA reductase was analyzed using an Elisa kit from SunLong Biotech Co., Ltd., Hangzhou, China. All procedures were conducted according to the manufacturer’s instructions.

### 2.8. ^1^H NMR Measurement, Data Processing and Multivariate Analysis

NMR measurement was conducted based on Abu Bakar Sajak et al. [18] using a 500 MHz Varian INOVA NMR spectrometer (Varian Inc., Palo Alto, CA, USA) functioning at 499.92 MHz. In brief, the supernatant from the thawed urine was mixed in a 2:1 ratio to the phosphate buffer solution (KH_2_PO_4_, pH 7.4, 0.1% TSP) prepared in deuterium oxide (D_2_O). The mixture was then transferred into a 5 mm NMR tube and subjected to NMR analysis. A proton NMR was first conducted in each sample, followed by a standard water-suppression one dimensional NMR, PRESAT sequence with 64 scans and acquisition time 206 s.

The spectral processing (phasing and binning) was performed based on consistent setting using Chenomx software (v. 8, Edmonton, AB, Canada), with TSP set as the reference peak (chemical shape indicator) at δ 0.00. A binning process set to δ 0.04 per bin was performed on each spectrum excluding urea (δ 5.645–5.92) and the water region (δ 4.75–4.98), which produced a total number of 227 bins. Each of these points was lined using the total area normalization.

### 2.9. Statistical Analysis (Multivariate and Univariate Analysis)

The preprocessed NMR dataset was analyzed with SIMCA-P software multivariate data analysis (v. 14, Umetrics, Umea, Sweden). A non-supervised data analysis was first performed to visualize and to discriminate the group. The partial least square analysis (PLS), a supervised data analysis, was also carried on the NMR spectral data to determine the relationship between the metabolites with the biochemistry and the HMG-CoA reductase activity. The validation of the model was described by the goodness of fit, where differences between *R2* value and *Q2* value should not be more than 0.3, and the cross-validation parameter for *Q2* and *R2* [25].

The numerical data from in vitro and in vivo experiments were presented as the mean ± standard deviation. Since the sample sizes were unequal by the end of the study, the statistical differences of serum biochemistry and hydroxymethylglutaryl CoA (HMG-CoA) reductase assay were evaluated using one-way analysis of variance (ANOVA) with Tukey–Kramer test as the post hoc (for variables that have unequal sample sizes and have equal variance: AST, ALT and TC) or Welch’s test and Games–Howell as post hoc (for variables that have unequal sample sizes and have unequal variance: HDL-C, LDL-C). For the relative quantification of the metabolites level, Welch’s test (a modified version of Student T-test) was used to determine the two groups’ significant level. The confidence interval was set at 95%, and *p* < 0.05 was considered significant.

## 3. Results

### 3.1. Characterization of PRM

Based on our previous study, the proton NMR of PRM containing primary and secondary metabolites includes amino acids, sugars, 5-hydroxymethylfurfural (5-HMF) and ascorbic acid (Vitamin C). The nutritional composition of PRM reveals it contained about 43.40% carbohydrates, whereby 18% of it came from fructose [21]. The high content of sugar can mask the appearance of other secondary metabolites in spectra. Thus, the determination of ascorbic acid was conducted using the titration method, and removal of sugar was undertaken to determine hydrophobic and aromatic compounds of PRM before HPLC. The results of ascorbic acid content showed that PRM contained 5.06 ± 0.14 mg of ascorbic acid in each 100 mL of PRM, equivalent to 15.14 ± 0.43 µg of ascorbic acid in 500 mg of PRM. 

For quantification purposes, only PRM acidified and ethyl acetate fractions were chosen for quantification purposes (Supplementary Materials, Figures S1 and S3). This is because for baseline, intensity and separation of the chromatogram peaks from these two samples were better than the others (Supplementary Materials, Figures S2 and S4). The chromatogram of the acidified PRM (Supplementary Materials, Figure S1) showed the presence of 5-HMF. The removal of 5-HMF using SPE improved the chromatogram (Supplementary Materials, Figure S2). There are several peaks on the chromatogram. However, we only managed to identify 2 peaks in PRM after spiking with the standards in HPLC. The quantification of these two peaks was made in ethyl acetate fraction. This sample (PRM after SPE) was also sent for LC-MS to detect the other compounds. The LC-MS chromatogram showed that PRM after SPE contains primary and secondary metabolites such as fatty acids, phenolic compounds, terpenoids, phospholipid and sterol (Supplementary Materials, Figure S5 and Table S1). 

The ethyl acetate result (Supplementary Materials, Figure S3) showed the presence of 2 major peaks, which are 6-gingerol and 6-shogaol. From the quantitative analysis, each 500 mg of PRM contains 8.21 ± 0.44µg of 6-gingerol and 45.06 ± 2.57µg of 6-shogaol (Table 1).

### 3.2. Serum Biochemistry and Inhibition of HMG-CoA Reductase Activity

Liver function tests such as aspartate aminotransferase (AST) and alanine aminotransferase (ALT) have been used to monitor and diagnose a liver disease or damage [26]. Even though the result for serum biochemistry (Table 2) shows that high cholesterol diet consumption increased the AST and ALT level, no significant differences (*p* > 0.05) were noted when compared to the other group. Increased levels of AST and ALT were also noted in the PRM treated groups and D group. Nevertheless, the only significant increase (*p* < 0.05) was noted in the AST level in the PRM-M and D groups when compared to the C group. Previously, elevated AST and ALT levels have been recorded in hypercholesterolemia and hyperlipidemia, herbal, and statin studies [21,26,27]. The consumption of high cholesterol diet and drugs can influence the liver’s rate of clearance. 

The consumption of a high cholesterol diet also increased the total cholesterol and low-density lipoprotein (LDL-C) and decreased high-density lipoprotein (HDL-C). However, among the PRM treated groups, PRM in high doses (500 mg/kg body weight) and medium doses (250 mg/kg body weight) managed to significantly (*p* < 0.05) reduce the total cholesterol compared to the HC group. A similar trend was also noted in LDL-C, where the PRM-H, PRM-M and D groups managed to significantly (*p* < 0.05) reduce the LDL-C level compared to the HC group. 

This result is also supported by hydroxymethylglutaryl CoA reductase activity (HMG- CoA reductase). The highest inhibition activity (the lowest activity of HMG-CoA reductase) can be seen in the control group, followed by the simvastatin group (D), PRM-H, PRM-M, PRM-L and finally, the hyperlipidemia group (HC). HMG- CoA reductase is a rate-limiting enzyme involved in the mevalonate pathway, which requires cholesterol and isoprenoid production. Thus, controlling the HMG-CoA reductase enzyme has been one of the approaches to control cholesterol levels [28].

### 3.3. ^1^H-NMR Spectral Analysis of Urine Samples

A total of 21 metabolites (Supplementary Materials, Figure S6) were identified using the Chenomx profiler consisting of primary metabolites such as amino acids and intermediates in the tricarboxylic acid (TCA) cycles, organic acids and others. To better understand the effect of PRM in the induced high lipidemia rats, multivariate analysis (MVDA) was conducted on the binning spectral data. In addition, an unsupervised analysis, principal component analysis (PCA), was first performed to overview and visualize the pattern.

The first PCA model (M1) that included all six groups showed clear discrimination between C, HC and D (Supplementary Materials, Figure S7). However, no clear discrimination can be seen between the different doses due to the variabilities of the data. The model consists of 3 principal components, where the first total variance for the first two components was 69.14% and *R2X*_(cum)_ and *Q2*_(cum)_ > 0.5. From PC1, the metabolites such as creatinine, TCA intermediates (citrate, 2-oxoglutarate and succinate), isoleucine and phenyl acetyl glycine were high in the non-treated, C and HC groups. In contrast, metabolites such as mannose, trigonelline, fumarate, formate, methyl nicotinamide (MNA) and pyridoxine were high in the treated groups (PRM-H, PRM-M, PRM-L and D). The discrimination between C and HC groups can be noted from PC2, where the high metabolites in HC include trimethylamine-*N*-oxide (TMAO), succinate, mannose, allantoin, fumarate and dimethylglycine (DMG).

**Figure 1:**
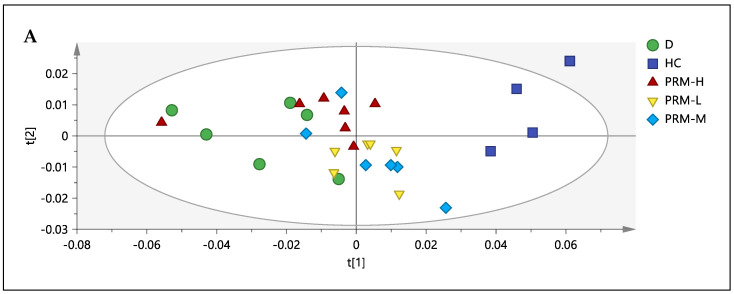

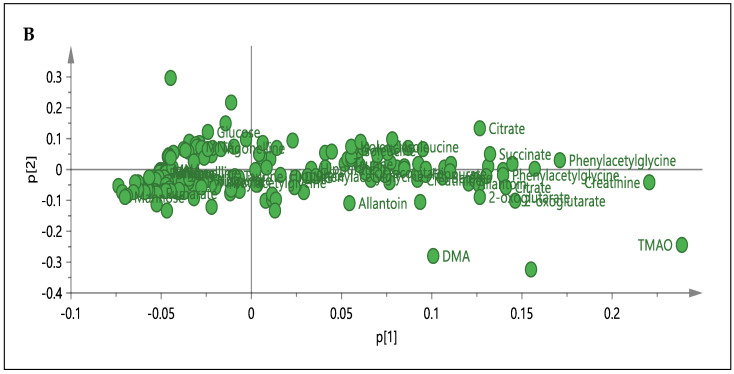
Principle component analysis (PCA) M2. Score plot (**A**) and loading scatter plot (**B**) obtained using ^1^H-NMR from simvastatin (D), hyperlipidemia (HC), polyphenol-rich mixture high dose, 500 mg/kg (PRM-H), polyphenol-rich mixture medium dose, 250 mg/kg (PRM-M) and polyphenol-rich mixture low dose, 150 mg/kg (PRM-L).

Since no clear separation can be seen between the doses on M1, another PCA model, M2 (Figure 1), was built comprising all the groups, excluding the normal control (C) group. The C group was removed to see the effect of the treatment itself and to get a better separation among the treated group (by excluding normal control can reduce the variabilities in the data) by comparing it with the HC group.

A new model with three principal components and *R2X*_(cum)_ and *Q2*_(cum)_ > 0.5 was achieved with the differences between *R2X*_(cum)_ and *Q2*_(cum)_ being less than 0.3. A variance of 67.69% could be explained from the first two components. From the score scatter plot, HC can be discriminated from the D group (all six rats) and PRM-H group (six rats out of seven rats) from PC1. In PC1 (58.40% variance), metabolites such as trimethylamine-*N*-oxide (TMAO), creatinine, betaine, hippurate, cis-aconitate, phenyl acetyl glycine, TCA intermediates (2-oxoglutarate, succinate and citrate), amine derivatives/by-product (dimethylamine (DMA)) and amino acid (isoleucine) were higher in the HC group compared to D and PRM-H groups.

Meanwhile, metabolites such as mannose, fumarate, methyl nicotinamide (MNA), trigonelline and pyridoxine were high in D and PRM-H groups. Only 9.29% can be explained from PC2, whereby the metabolites were high in PRM-H (six rats out of seven rats), and D (five rats out of six rats) were glucose and isoleucine. In contrast, metabolites such as TMAO, mannose and fumarate were high in the lower side of the quadrant, representing primarily for the PRM-M group (four rats out of six rats) and PRM-L dose (four rats out of six rats). From the separation, it can be deduced that among the different doses, PRM-H had a better result compared to the others as it was separated from the HC group by PC1 and the PRM-H located in the same quadrant as the D group (simvastatin). It was also noted that PRM-M and PRM-L did not regulate TMAO levels, the primary metabolites that contribute to separation between HC and C from the M2 model. Thus, PRM-H was chosen for correlation and relative quantification, even though there were similarities in serum biochemistry results for PRM-H and PRM-M. The group discrimination in urine metabolomics was based on the whole metabolites, whereas the serum biochemistry was only limited to specific parameters.

### 3.4. Correlation between the Metabolites, HMG-CoA Reductase Activity and Low-Density Lipoprotein

**Figure 2:**
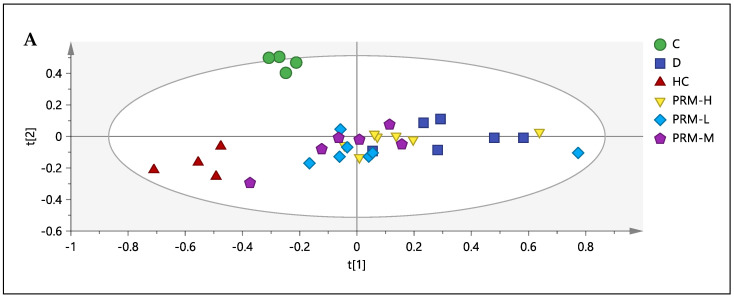

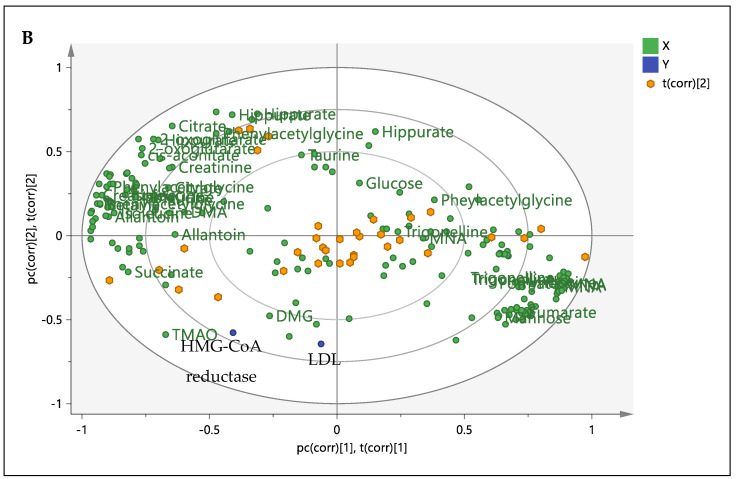
Partial least square (PLS) M3. Score plot (**A**) and biplot (**B**) obtained using ^1^H-NMR from control (C), simvastatin (D), hyperlipidemia (HC), polyphenol-rich mixture high dose, 500 mg/kg (PRM-H), polyphenol-rich mixture medium dose, 250 mg/kg (PRM-M) and polyphenol-rich mixture low dose, 150 mg/kg (PRM-L).

For correlation and relationship purposes, a supervised method, partial least square (PLS) in MVDA, was applied to the binned spectral data (assigned as X-block) with the biochemistry and HMG-CoA reductase activity (assigned as Y-block). In this model, the chemical shifts (binned spectral data) from the best dose, PRM-H, were presented by X-block as predictor variables. In contrast, the response variables were presented as Y-variable (biochemistry and HMG-CoA reductase activity). Thus, this model determines the metabolites that directly or indirectly affected the separation or contributed to the response variables. Among the biochemistry profile and HMG-CoA reductase, only LDL-C and HMG-CoA reductase activity gave a significant relationship with the model. A PLS model (Figure 2) with 3 components and *R2X*_(cum)_ = 0.72, *R2Y*_(cum)_ = 0.71 and *Q2*_(cum)_ = 0.43 was established. The discrimination and clustering in the PLS (M3) were almost similar to the PCA model of M1. The biplot showed that TMAO, dimethylglycine (DMG), succinate and allantoin were among the metabolites that contribute to HMG-CoA reductase activity and LDL-C level.

### 3.5. Relative Quantification of Metabolites and Pathway Analysis

A relative quantification of metabolites (Table 3) using univariate analysis (Welch’s Test) was done for C, HC, PRM-H and D groups based on the metabolites that contributed to the clustering in the PLS models (M3), with variance importance projection (VIP) values more than 0.5. A total of 20 metabolites were selected. The highest VIP values (>3.5) can be seen from trimethylamine-*N*-oxide (TMAO) from VIP 1 and VIP 2 values and citrate from VIP 2 values. Out of 18 metabolites, five were higher in the HC group that significantly (*p* < 0.05) differentiated HC from the C group. A total number of 16 and 15 metabolites significantly (*p* < 0.05) differentiated between PRM-H and D groups compared to the HC group, respectively. Interestingly, it was noted that the changes of metabolites in PRM-H and D against the HC group were more or less similar to each other, and this result supports the previous MVDA results.

**Figure 3:**
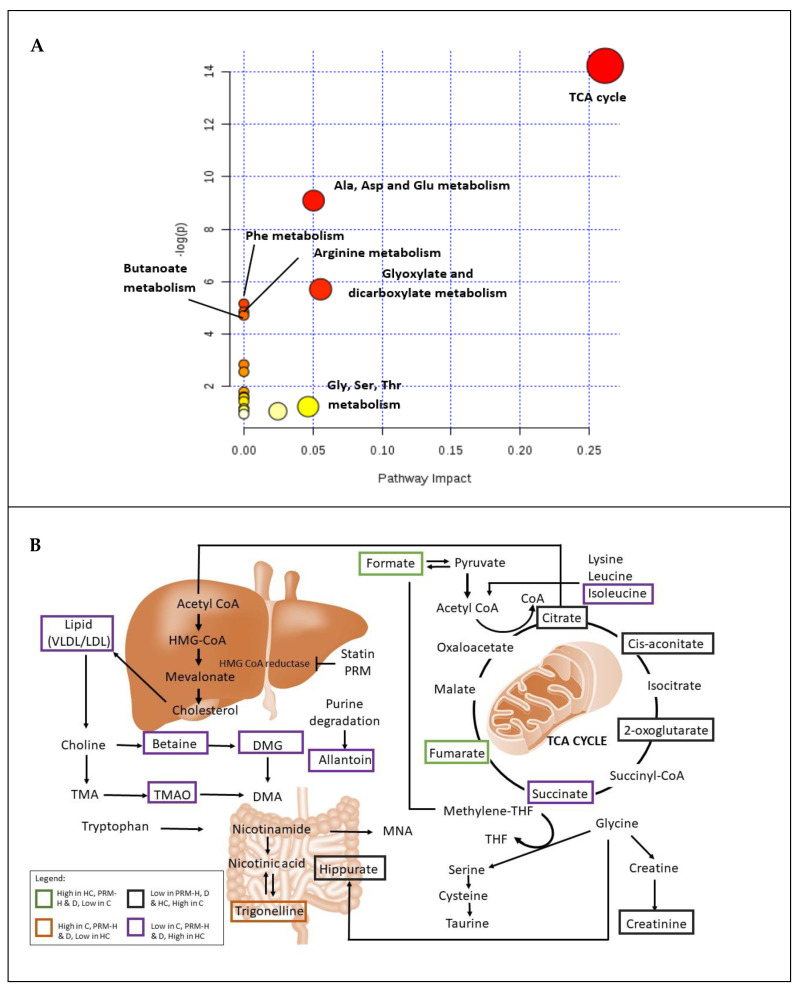
Illustrated pathways involved. (**A**) Summary from MetaboAnalyst 4.0 pathway analysis. The color and the size of the nodes represent the pathway’s degree of importance, whereas the large red notes represent the highest level of change in rats treated with PRM. Moderate, slight and zero importance is represented by the colors orange, yellow and white. The X-axis in the pathway analysis represents the hits number of metabolites in each pathway, while the Y-axis represents the number of -log (p) of the metabolites. (**B**) Tentative disturbed metabolism and pathways in Control (C), hyperlipidemia (HC), polyphenol-rich mixture high dose (PRM-H) and simvastatin (D). TMA: Trimethylamine; TMAO: Trimethylamine-N-oxide; HMG CoA: Hydroxymethylglutaryl-CoA; DMG: Dimethylglycine; DMA: Dimethylamine and THF: Tetrahydrofolate.

For pathway analysis for PRM in high cholesterol rats, only the significant metabolites were chosen for pathway analysis using MetaboAnalyst 4.0 [29]. The generated data (Figure 3A and Supplementary Material, Table S2) showed that 17 pathways were changed by PRM-H treatment. The most hits were found in carbohydrate metabolism, with TCA cycle (5 hits), followed by amino acids metabolism, with alanine, aspartate and glutamate metabolism (4 hits) and glyoxylate and dicarboxylate metabolism (3 hits). The importance of the metabolites in the network was shown as the pathway impact factor, where the TCA cycle (0.26) had the highest impact factor. The disturbed pathways and metabolisms were also illustrated in Figure 3B. This result is vital in giving a complete picture and understanding of the pathways that change by PRM-H intervention in hyperlipidemic rats.

## 4. Discussion

The NMR spectra and chromatograms of PRM showed compounds such as ascorbic acid, 5-HMF, 6-gingerol and 6-shogaol. A previous study showed that ascorbic acid could improve hyperlipidemia and cardiac function in streptozotocin-diabetes rats [30]. At the same time, gingerol and shogaol showed plasma lowering activity in hamsters by increasing sterol secretion [31]. Furthermore, they also inhibit LOX-1 (invoked by oxidized LDL) [32] and upregulate LDLR protein and cholesterol efflux-related genes LXRα and ABCA1 in HepG2 cells [28]. Furthermore, gingerol and shogaol were revealed as good HMG-CoA inhibitors [33], reflected in our HMG-CoA inhibition result. In addition, 5-HMF has also been shown as a good antioxidant and managed to improve lipid profile and malondialdehyde (MDA) in liver tissue of alcoholic liver oxidative injury in mice [34]. These findings are in line with the biochemistry results, where there was an improvement in the lipid profile of the hyperlipidemic rats, especially in total cholesterol and LDL-C. 

### 4.1. Carbohydrate Metabolism

This study found a lower concentration of TCA intermediates metabolites in all high cholesterol-fed diets than in the C group. This finding was in line with a previous study using a high-cholesterol diet by Jiang et al. [35], who noted the downregulation in 2-oxoglutarate, succinate and citrate levels. The low availability of dissolved oxygen in the body due to hyperlipidemia can delay the conversion of pyruvate to acetyl-CoA, affecting TCA intermediates. Lower TCA intermediates in PRM-H and D groups compared to the C group indicate changes in the energy metabolism dynamics. In addition to the hyperlipidemia condition, lower TCA intermediates might also be associated with the HMG-CoA reductase inhibition by PRM-H and D (simvastatin). Previously, statin has been noted to regulate both glucose and lipid homeostasis. Through inhibition of HMG-CoA reductase, statin indirectly activates sterol regulatory element-binding protein 2 (SREBP-2), controlling glucose transporter (especially GLUT-2 in hepatocyte) and enzymes such as glucokinase (GCK) [36,37]. The reduction in glucose transportation affects the production of glucose-6-phosphate (via glycolysis) and the other precursor associated with the TCA cycle, which explains the lower TCA intermediates in the urine of the D group. The same trend also can be found in the PRM-H group due to the presence of 6-gingerol. It has been noted that 6-gingerol can activate SREBP-2 [16], whereby SREBP-2 plays a significant role in the regulation of glucose and lipid metabolism [38].

### 4.2. Amino Acid Metabolism

Amino acids are essential for cellular building, reparation of damaged cells and lipid biosynthesis [39]. Branch chain amino acids (BCCAs) such as leucine and isoleucine has been linked to metabolic syndromes such as obesity and diabetes [40]. According to Yang et al. [41], there is a positive correlation between BCAAs and dyslipidemia levels. At the same time, it also has an inversely proportionate correlation with high-density lipoprotein (HDL-C). From our study, a lower level of isoleucine was noted compared to the HC group. This result is also supported by the lower level of low-density lipoprotein (LDL-C) in the serum compared to the HC group.

The relationships between alanine, aspartate and glutamate metabolism and glycine, serine and threonine metabolism and high cholesterol are yet to be understood. The 2 hits out of 34 metabolites in glycine, serine and threonine metabolism and 4 hits out of 28 metabolites in alanine, aspartate and glutamate were from the TCA intermediates (i.e., fumarate, cis-aconitate and succinate). The impact of these metabolites proved that the changes in carbohydrate metabolism could also affect amino acid metabolism.

A study by Sookian and Pirola [42] found an association of abnormalities in glutamate metabolism and the pathogenesis of metabolic syndrome linked with aminotransferase reactions in the liver. They also postulate that abnormal levels of liver enzymes (AST and ALT) can reflect the level of hepatic transamination of amino acids in the liver. As we all know, the liver plays a significant role in producing and clearance of cholesterol. Thus, our result suggests that PRM not only alters carbohydrate metabolism, but it also affects liver homeostasis. Nevertheless, it needs to be noted that the administration of drugs and herbs can affect the level of AST and ALT, as the liver needs to clear or detoxify the compound from the system.

Meanwhile, for tyrosine metabolism, the precursor can come from phenylalanine through enzymatic and or non-enzymatic degradation of free radicals in phenylalanine metabolism [43]. Changes in microbial composition in gut microbiota are usually reflected in tyrosine and phenylalanine metabolism [44]. Phenylalanine converted to trans cinnamate acid by phenylalanine ammonia-lyase. It will enter the hippurate pathway as benzoate [45]. The addition of benzoate from trans cinnamate acid and degradation of hippurate can increase the availability of benzoate entering butanoate metabolism, which is later involved in intestinal immunity and gut microbiota [46]. Therefore, the low hippurate level in PRM-H and D groups indicated an alteration in the gut microbiota community. The changes in gut microbiota are discussed in detail in the next section.

### 4.3. Gut Microbiota and the Other Metabolism

Metabolic disturbance in gut microbiota has been linked to the progression of metabolic syndromes such as obesity and diabetes [47]. Hippurate level in urine can be an indicator of the degradation of dietary fiber by the gut microbiota. Low hippurate levels in PRM-H and D groups indicate that (1) PRM-H contained low dietary fiber, and (2) PRM-H and statin might inhibit the gut microbiota biome. Apart from that, this trigonelline, a by-product of niacin metabolism (Vitamin B3), also might contribute to this result. Trigonelline in this study is significantly higher (*p* < 0.05) compared to the HC group. Anwar et al. [48] reported that the trigonelline could inhibit intestinal microflora involved in choline metabolism. These gut microbiota (i.e., Citrobacter genus) from choline metabolism can produce trimethylamine (TMA), which is later converted to trimethylamine-*N*-oxide (TMAO) by flavin monooxygenase (FMO_3_).

A high level of TMAO has been linked to an increased risk of atherothrombotic cardiovascular disease. TMAO can promote foam cells formation by inducing macrophage deregulating enterohepatic cholesterol and bile acid metabolism and impairing macrophage reverse cholesterol transport (RCT). Reduction in TMAO is also associated with a better lipid profile (total cholesterol, LDL and triglyceride) compared to the high fat-induced C57BL/6J mice via restoration autophagy [49]. These changes also highlighted the association between trigonelline, TMAO and lipid profile. Our results showed that PRM-H, which contains higher trigonelline, has a lower TMAO level and a better lipid profile.

Metabolic syndrome is also regularly associated with oxidative stress. Allantoin is considered an oxidative stress biomarker produced through the non-enzymatic process [8]. In this study, the urinary level of allantoin in the PRM-H was significantly reduced compared to the hyperlipidemia group (HC), which indicate that PRM-H improved the oxidative stress level in the hyperlipidemic rats.

### 4.4. Study Limitations and Future Recommendations

From HPLC chromatograms of the samples, only three compounds (5-HMF, 6-shagaol and 6-gingerol) were identified and quantified after comparing the RT and spiking with the standards. Further identification of the unknown should be made using MS/MS to see the mass and the fragmentation ions of the unknown compounds. 

The metabolites affected by the PRM treatment was successfully identified using urine metabolomics. However, confirming these affected pathways to exploit their further potential as biomarkers needs to be done. Combining information with other omics such as proteomics can give a complete picture for future research, primarily targeting a particular pathway. In addition, a targeted approach in vivo (applying techniques such as CRISPR) also can be made to confirm the pathway and the metabolites that can be the biomarkers to the disease.

## 5. Conclusions

In summary, both high dose (PRM-H, 500 mg/kg) and medium-dose (PRM-M 250 mg/kg) of PRM managed to lower the total serum cholesterol and low-density lipoprotein level in hyperlipidemic rats. However, at the level of the metabolites, multivariate analysis revealed that the changes of metabolites in PRM-H were better than PRM-M as they managed to reduce the primary biomarker in hyperlipidemic rats, TMAO. In addition, discrimination in the multivariate analysis also implies that the PRM-H effect on hyperlipidemic rats is more or less similar to the standard drug, simvastatin, which suggested the mechanism of PRM is equivalent to simvastatin. Pathway analysis using MetaboAnalyst 4.0 managed to reveal 17 pathways that the PRM-H has altered. The highest impact pathway can be seen in carbohydrate metabolism, especially in TCA cycles. Alteration of metabolite level can also be seen in amino acid metabolism, as most of the precursors from TCA intermediates also involve amino acid metabolism. At the same time, changes in gut microbiota can be seen from the hippurate and trigonelline levels, affecting metabolisms such as propanoate and butanoate. This study also highlighted the relationships between serum biochemistry of LDL-C and HMG-CoA reductase with urine metabolites (trimethylamine-*N*-oxide, dimethylglycine, allantoin and succinate).

## Figures and Tables

**Table 1:** Quantitative determination of compounds in PRM.

No	Sample	RT	Identified Compound	Weight (µg/500 mg of PRM)
1	PRM acidified	4.84	5-Hydroxymethyfurfural (5-HMF)	1126.77 ± 21.08
2	Ethyl acetate fraction	25.76	6-gingerol	8.21 ± 0.44
2	Ethyl acetate fraction	31.26	6-shogaol	45.06 ± 2.57

RT is abbreviation for retention time.

**Table 2:** Serum biochemistry and hydroxymethylglutaryl CoA (HMGCoA) reductase activity for control, polyphenol-rich mixture (PRM) treated rats and drug (simvastatin).

Test	AST	ALT	Chol	Tgl	LDL-C	HDL-C	HMGCoA Reductase Activity (pg/mL)
Group	U/L	U/L	mmol/L	mmol/L	mmol/L	mmol/L	
Control (C)	66.50 ± 45.96	78.50 ± 58.69	1.65 ± 0.21 ^#^	1.04 ± 0.03	0.16 ± 0.00 ^#^	1.46 ± 0.23	241.61 ± 44.48 ^#^
Hyperlipidemia (HC)	111.00 ± 28.87	144.00 ± 40.41	2.21 ± 0.19	0.82 ± 0.17	0.89 ± 0.11	1.16 ± 0.05	389.71 ± 64.92 *
PRM High Dose (PRM-H)	106.75 ± 17.73	87.75 ± 29.92	1.55 ± 0.17 ^#^	0.76 ± 0.27	0.48 ± 0.10 * ^#^	1.21 ± 0.12	291.93 ± 20.19 ^#^
PRM Medium Dose (PRM-M)	138.60 ± 15.51 *	61.80 ± 33.12	1.74 ± 0.17 ^#^	0.85 ± 0.16	0.56 ± 0.14 * ^#^	1.28 ± 0.11	309.61 ± 63.12 ^#^
PRM Low Dose (PRM-L)	126.50 ± 22.19	79.75 ± 18.28	1.85 ± 0.05	0.78 ± 0.24	0.64 ± 0.15 *	1.31 ± 0.06 ^#^	372.81 ± 34.49 *
Simvastatin (D)	148.50 ± 30.44 *	85.00 ± 56.00	1.70 ± 0.25 ^#^	0.97 ± 0.21	0.64 ± 0.18 *	1.15 ± 0.12	241.16 ± 56.60 ^#^

AST stands for aspartate aminotransferase, ALT for alanine aminotransferase, Chol for total cholesterol, Tgl for triglyceride, LDL for low-density lipoprotein and HDL for high-density lipoprotein. * Indicates significant differences (*p* < 0.05) with the control group (C), while ^#^ indicates significant differences (*p* < 0.05) with the hyperlipidemia group (HC). The differences between the groups were tested using One Way ANOVA with Tukey–Kramer test (for variables that have unequal sample sizes and have equal variance: AST, ALT and TC) as post hoc or Welch’s test and Games–Howell as post hoc (for variables that have unequal sample sizes and have unequal variance: TG, HDL-C and LDL-C).

**Table 3:** Relative quantification of metabolites level based on binning spectral data of ^1^H NMR in control (C), hyperlipidemia (HC), polyphenol-rich mixture high dose (PRM-H) and simvastatin (D) groups.

Metabolites	δ ^1^H (ppm)	VIP		HC vs. C	PRM-H vs. HC	D vs. HC
		1	2			
Trimethylamine-*N*-oxide (TMAO)	3.26	6.75	4.92	H ***	L **	L **
Creatinine	3.02	3.06	2.18	L	L ***	L ***
Phenylacetylglycine	3.66	1.71	2.89	L	L **	L ***
2-oxoglutarate	2.42, 2.98	0.39	2.88	L **	L **	L **
Succinate	2.38	3.20	1.99	H	L **	L **
Citrate	2.66	1.20	3.67	L	L *	L ***
Allantoin	5.38	1.36	1.25	H	L *	L ***
Dimethylamine (DMA)	2.70	1.10	1.34	L	L	L
Hippurate	3.94	1.04	1.92	L	L **	L ***
Isoleucine	1.22, 1.42	1.04	0.78	H *	L **	L **
Cis-aconitate	3.10	0.35	1.01	L *	L **	L *
Taurine	3.25	0.86	1.14	L	L	L *
Betaine	3.89	1.56	1.06	H	L **	L **
Fumarate	6.51	0.61	0.55	H	H ***	H ***
Formate	8.44	0.31	0.74	H	H **	H
Dimethylglycine (DMG)	2.91	0.76	0.60	H *	L	L
Trigonelline	9.10, 8.82	0.39	0.76	L	H **	H
Pyridoxine	7.66	0.10	0.54	L	H	H
Methylnicotinamide (MNA)	9.26, 8.94, 8.86	0.42	0.86	H	H **	H **
Mannose	5.17	0.78	0.68	H	H *	H *

The * denotes the significant level when comparing between the two groups, whereby * *p* < 0.05, ** *p* < 0.01 and *** *p* < 0.001 using Welch’s Test.

## Data Availability

The data presented in this study are available in the Supplementary Materials.

## Supplementary Materials

The following are available online at https://www.mdpi.com/article/10.3390/nu13103573/s1. Figure S1: Representative chromatogram of acidified PRM at 5 mg/mL, where A is a chromatogram at 214 nm, B is a chromatogram at 280 nm, and C is a chromatogram at 360 nm. Figure S2: Representative chromatogram of PRM at 1 mg/mL after solid-phase extraction, where A is a chromatogram at 214 nm, B is a chromatogram at 280 nm, and C is a chromatogram at 360 nm. Figure S3: Representative chromatogram of PRM ethyl acetate fraction at 12.5 mg/mL, where A is a chromatogram at 214 nm, B is a chromatogram at 280 nm, and C is a chromatogram at 360 nm. Figure S4: Representative chromatogram of PRM aqueous fraction, where A is a chromatogram at 214 nm, B is a chromatogram at 280 nm, and C is a chromatogram at 360 nm. Figure S5: Total ion chromatogram (TIC) of PRM after solid phase extraction. Figure S6: Urine spectra representative from 0.5–10.2 ppm. Label: 1: Isoleucine, 2: Succinate, 3: 2-Oxoglutarate, 4: Citrate, 5: Dimethylamine (DMA), 6: N, N Dimethylglycine (DMG), 7: Creatinine, 8: Cis-aconitate, 9: Taurine, 10: N-Phenylacetylglycine, 11: Hippurate, 12: Trigonelline, 13: 1-Methylnicotinamide (MNA), 14: Glucose, 15: Mannose, 16: Allantoin, 17: Fumarate, 18: Pyridoxine, 19: Formate, 20: Trimethylamine-N-oxide (TMAO) and 21: Betaine. Figure S7: Principal component analysis PCA, M1. Score Plot (A) and Loading Scatter Plot (B) obtained using 1H-NMR from control (C), simvastatin (D), hyperlipidemia (HC), herbal mixture high dose, 500 mg/kg (PRM-H), herbal mixture medium dose, 250 mg/kg (PRM-M) and herbal mixture low dose, 150 mg/kg (PRM-L). Table S1: Tentative compounds in PRM after solid phase extraction. Table S2: List of pathways involved using qualitative MetaboAnalyst 4.0.
