# Supplementary material for: The Changes in Endogenous Metabolites in Hyperlipidemic Rats Treated with Herbal Mixture Containing Lemon, Apple Cider, Garlic, Ginger, and Honey
Source: Nutrients. 2021 Oct 12;13(10):3573. doi: 10.3390/nu13103573 (PMC8539352; doi:10.3390/nu13103573)
Supplement: Supplementary file 1 [file nutrients-13-03573-s001.zip › nutrients-1314294-supplementary.pdf]

Supplementary Materials

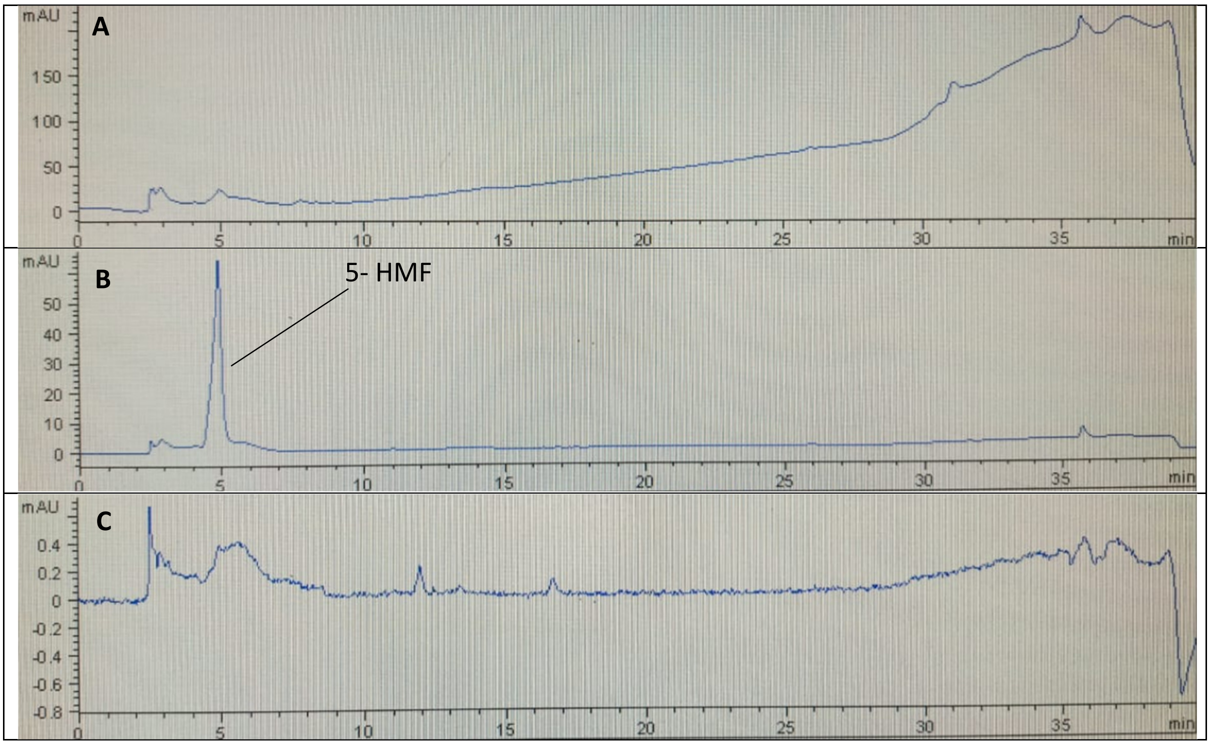

Figure S1. Representative chromatogram of acidified PRM at 5 mg/ml. Where A is a chromatogram at 214 nm, B is a chromatogram at 280 nm and C is a chromatogram at 360 nm.

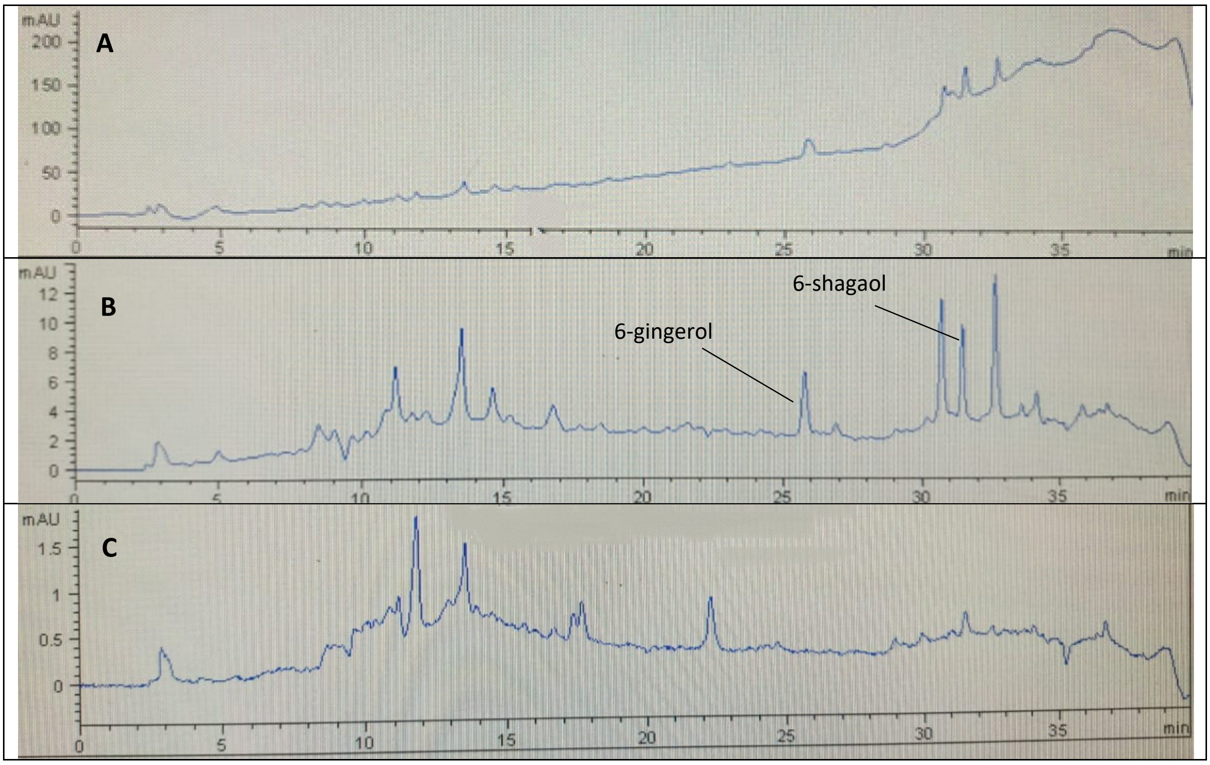

Figure S2. Representative chromatogram of PRM at 1 mg/ml after solid phase extraction. Where A is a chromatogram at 214 nm, B is a chromatogram at 280 nm and C is a chromatogram at 360 nm.

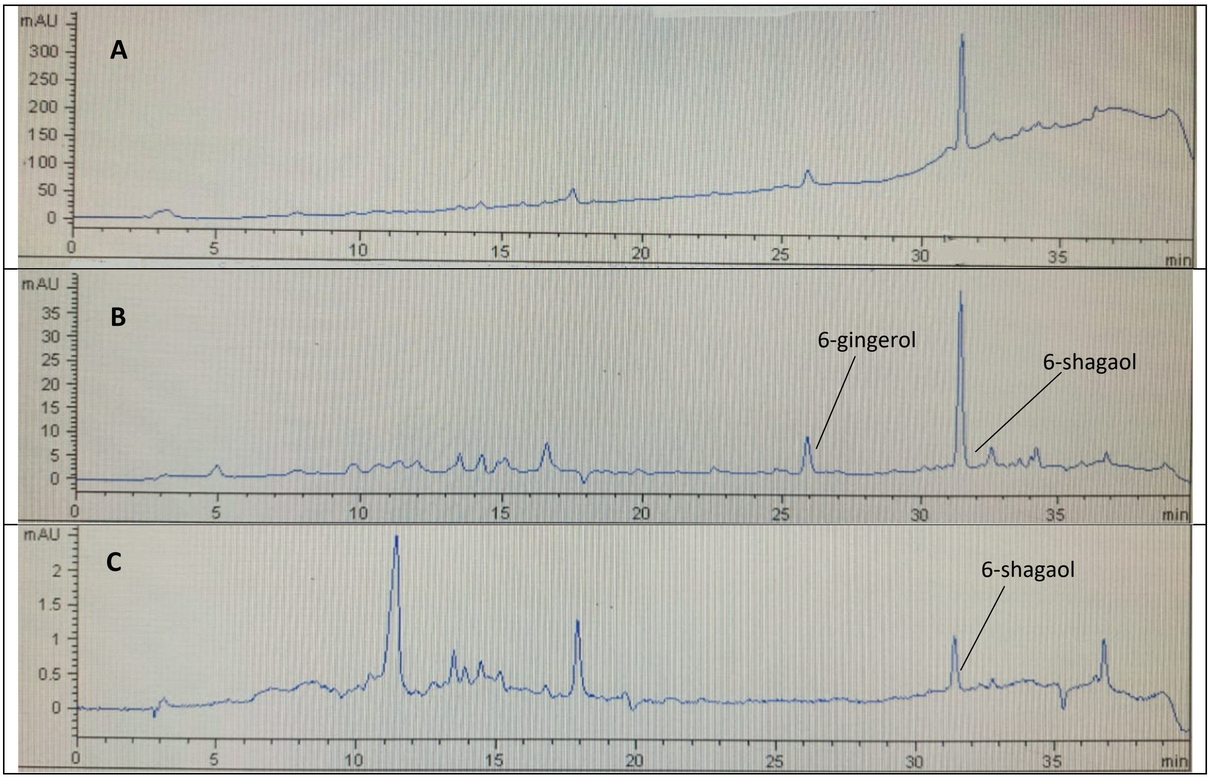

Figure S3. Representative chromatogram of PRM ethyl acetate fraction at 12.5 mg/ml. Where A is a chromatogram at 214 nm, B is a chromatogram at 280 nm and C is a chromatogram at 360 nm.

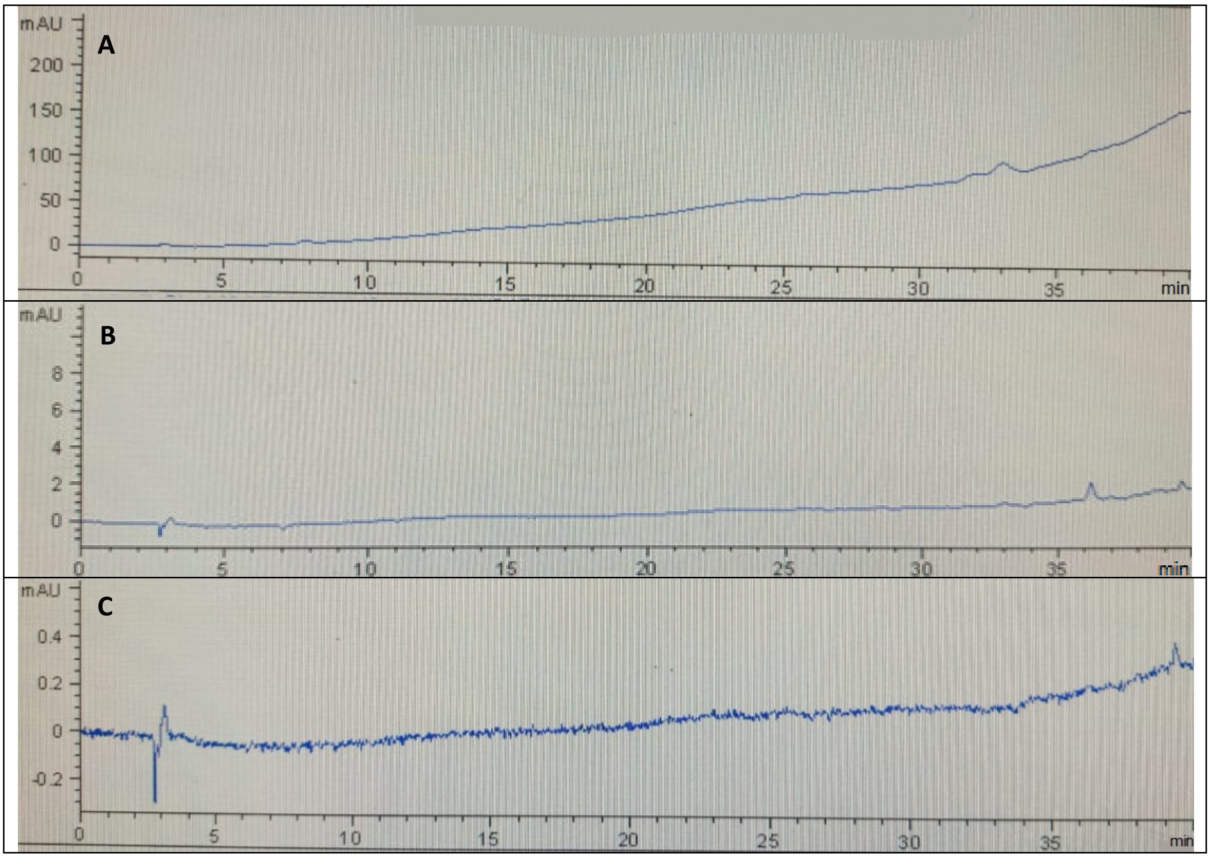

Figure S4. Representative chromatogram of PRM aqueous fraction. Where A is a chromatogram at 214 nm, B is a chromatogram at 280 nm and C is a chromatogram at 360 nm.

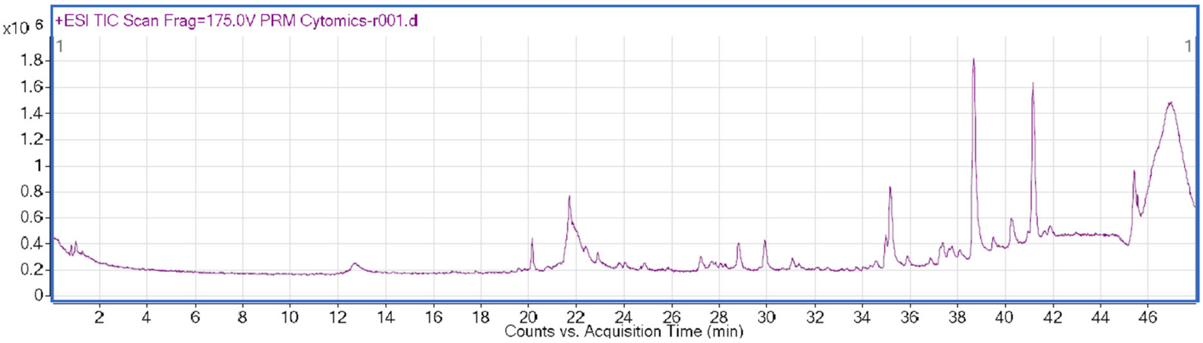

Figure S5. Total ion chromatogram (TIC) of PRM after solid phase extraction.

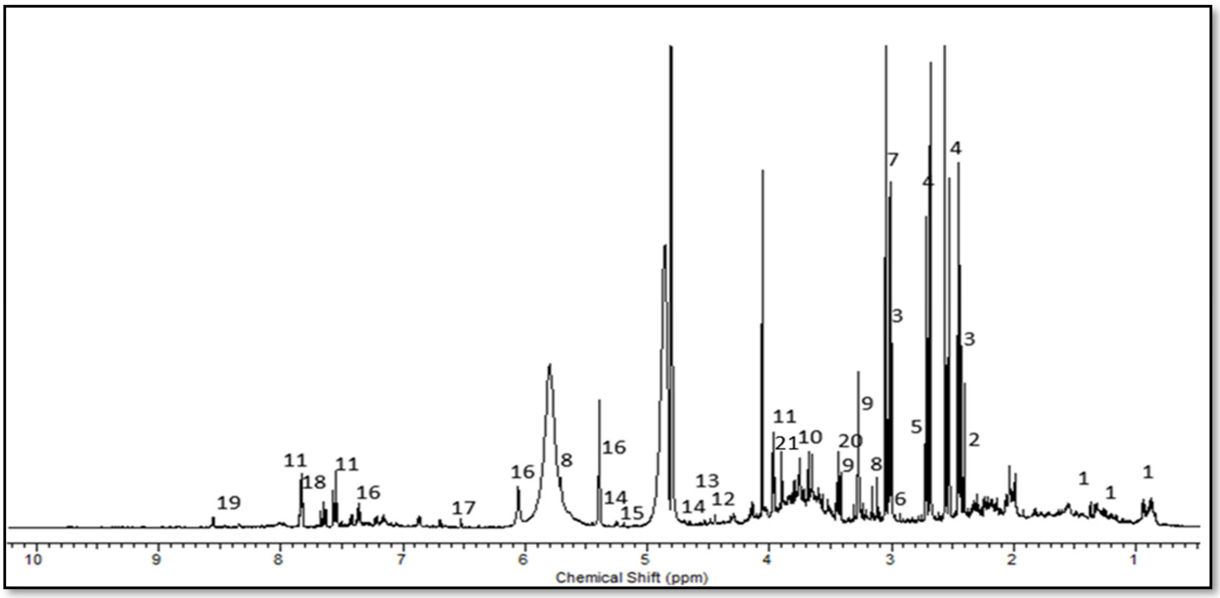

Figure S6. Urine spectra representative from 0.5 – 10.2 ppm. Label: 1: Isoleucine, 2: Succinate, 3: 2-Oxoglutarate, 4: Citrate, 5: Dimethylamine (DMA), 6: *N, N* Dimethylglycine (DMG), 7: Creatinine, 8: Cis-aconitate, 9: Taurine, 10: *N*-Phenylacetylglycine, 11: Hippurate, 12: Trigonelline, 13: 1-Methylnicotinamide (MNA), 14: Glucose, 15: Mannose, 16: Allantoin, 17: Fumarate, 18: Pyridoxine, 19: Formate, 20: Trimethylamine-*N*-oxide (TMAO) and 21: Betaine.

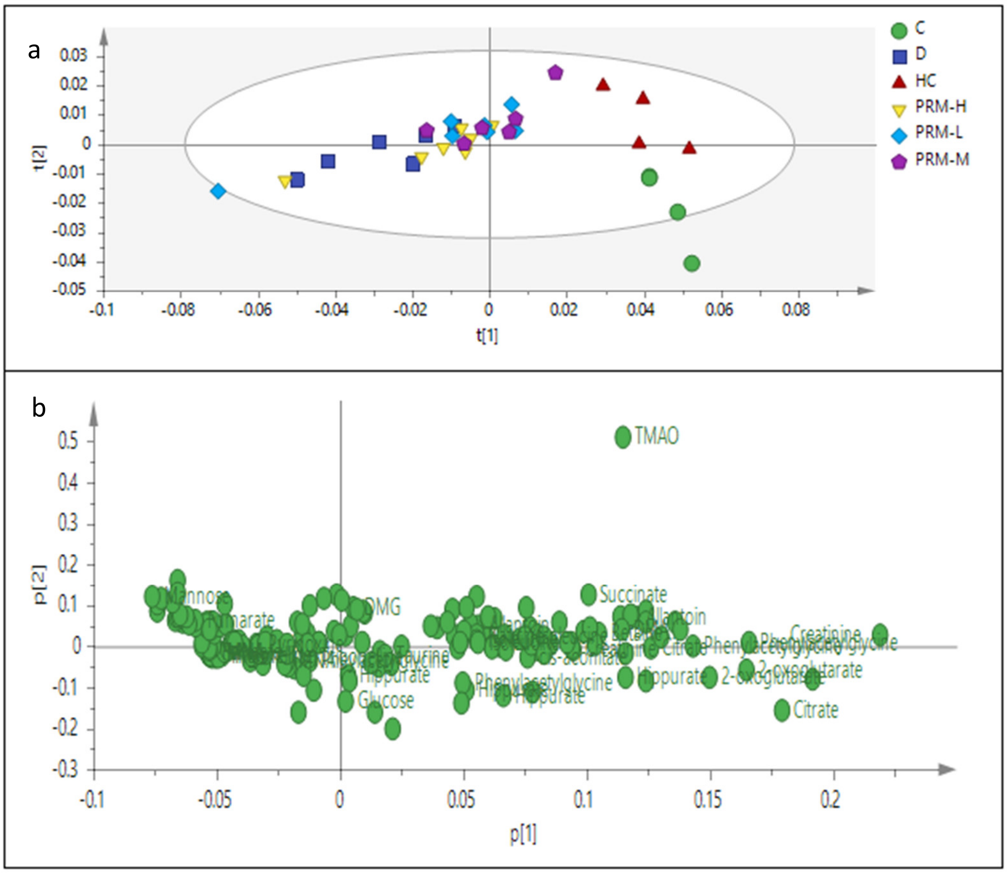

Figure S7. Principle component analysis PCA, M1. Score Plot (A) and Loading Scatter Plot (B) obtained using ^{1}H-NMR from control (C), simvastatin (D), hyperlipidemia (HC), polyphenol-rich mixture high dose, 500 mg/kg (PRM-H), polyphenol-rich mixture medium dose, 250 mg/kg (PRM-M) and polyphenol-rich mixture low dose, 150 mg/kg (PRM-L).

Table S1. Tentative compounds in PRM after solid phase extraction.

No	RT	Mass	Tentative Compound	Class
1	20.09	273.2663	C16 Sphinganine	Organonitrogen compound
2	34.03	255.2561	Palmitic amide	Fatty acid
3	29.90	278.1624	Leu Phe	Amino acid
4	23.75	294.1822	Gingerol	Phenolic compound
5	34.57, 35.16	330.2784	1-Monopalmitin	Fatty acid
6	35.16	312.2675	10-oxo-nonadecanoic acid	Fatty acid
7	35.16	256.2402	2-propyl-tridecanoic acid	Fatty acid
8	27.63	234.1614	Oxysolavetivone	Prenol lipids (sesquiterpenoids)
9	34.57	312.2664	10-oxo-nonadecanoic acid	Fatty acid
10	19.80	213.0815	2-(4-Methyl-5-thiazolyl)ethyl butanoate	Azole (Thiazoles)
11	33.12	545.4798	35-aminobacteriohopane-32,33,34-triol	Triterpenoids (Hopanoids)
12	24.79	652.4469	PA(P-16:0/18:4(6Z,9Z,12Z,15Z))	Phospholipids (Plasmalogen)
13	21.64	386.1706	Porson	Diarylheptanoids (Biphenyls)
14	24.01	414.2023	Armillaripin	Prenol lipids (Sesquiterpenoids)
15	28.74	576.4072	LysoPC(22:2(13Z,16Z))	Glycerophospholipids
16	31.06	482.3083	26-hydroxycholesterol 3-sulfate	Steroids and steroid derivatives

Table S2. List of pathways involved using qualitative MetaboAnalyst 4.0*

Rank		Total	Expected	Hits	% Found	Raw *p*	Impact
	(A) Carbohydrate Metabolism						
7	Citrate cycle (TCA cycle)	20	0.25	5	5.00	$6.61x10^{-7}$	0.26
6	Fructose and mannose metabolism	18	0.23	1	5.56	0.17	0
8	Pyruvate metabolism	22	0.28	1	4.55	0.20	0
10	Galactose metabolism	27	0.34	1	3.70	0.24	0
12	Glyoxylate and dicarboxylate metabolism	32	0.40	3	3.13	$3.32x10^{-3}$	0.06
14	Amino sugar and nucleotide sugar metabolism	37	0.47	1	2.70	0.31	0
	(B) Amino acid metabolism						
1	D-Glutamine and D-glutamate metabolism	6	0.08	1	16.67	$5.96x10^{-2}$	0
2	Valine, leucine and isoleucine biosynthesis	8	0.10	1	12.50	$7.70x10^{-2}$	0
3	Phenylalanine metabolism	12	0.15	2	8.33	$5.75x10^{-3}$	0
4	Arginine biosynthesis	14	0.18	2	7.14	$7.84x10^{-3}$	0
11	Alanine, aspartate and glutamate metabolism	28	0.35	4	3.57	$1.12x10^{-4}$	0.05
13	Glycine, serine and threonine metabolism	34	0.43	2	2.94	0.29	0.11
15	Valine, leucine and isoleucine degradation	40	0.50	1	2.50	0.33	0
16	Tyrosine metabolism	42	0.53	1	2.38	0.35	0.02
	(C) Propanoate and Butanoate metabolism						
5	Butanoate metabolism	15	0.19	2	6.67	$8.99x10^{-3}$	0
9	Propanoate metabolism	23	0.29	1	4.35	0.21	0
	(C) Translation						
17	Aminoacyl-tRNA biosynthesis	48	0.60	1	2.08	0.39	0

* The rank of each pathway was calculated based on the percentage of compounds found in each pathway (calculating hits of metabolites over the total number of metabolites). The number of expected metabolites was analyzed using hypergeometric testing to calculate the number of compounds expected to be in each pathway by chance alone. Raw *p* was calculated based on the number of hits and the total number of compounds in the pathway. The pathway impact represents the metabolite importance based on their position in the pathway, which determines by using relative betweenness centrality pathway topology analysis.
